# Adjunctive Clotiapine for the Management of Delusions in Two Adolescents with Anorexia Nervosa

**DOI:** 10.3390/bs11120173

**Published:** 2021-12-10

**Authors:** Jacopo Pruccoli, Giulia Joy Leone, Cristina Di Sarno, Luigi Vetri, Giuseppe Quatrosi, Michele Roccella, Antonia Parmeggiani

**Affiliations:** 1 IRCCS Istituto delle Scienze Neurologiche di Bologna, Centro Regionale per i Disturbi della Nutrizione e dell’Alimentazione in Età Evolutiva, U.O. di Neuropsichiatria dell’Età Pediatrica, 40138 Bologna, Italy; jacopo.pruccoli@studio.unibo.it (J.P.); giuliajoy.leone@studio.unibo.it (G.J.L.); cristina.disarno@studio.unibo.it (C.D.S.); 2 Dipartimento di Scienze Mediche e Chirurgiche (DIMEC), Università di Bologna, 40138 Bologna, Italy; 3 Oasi Research Institute-IRCCS, 94018 Troina, Italy; lvetri@oasi.en.it; 4 Department of Health Promotion, Mother and Child Care, Internal Medicine and Medical Specialties (PromISE), University of Palermo, 90128 Palermo, Italy; peppe.quatrosi@gmail.com; 5 Department of Psychology, Educational Science and Human Movement, University of Palermo, 90128 Palermo, Italy; michele.roccella@unipa.it

**Keywords:** clotiapine, anorexia nervosa, eating disorders, adolescents, inpatient treatment, antipsychotics

## Abstract

Clotiapine is an atypical antipsychotic indicated for the management of a series of acute psychotic disorders. The current literature lacks evidence concerning the tolerability and clinical use of this drug in the management of individuals with anorexia nervosa (AN). In this study, we report two cases of adolescents with AN, treated with clotiapine. The reason for the administration of clotiapine was, for both patients, the manifestation of bizarre delusions concerning food and calories. Patient 1 presented a presyncope after the first dose of clotiapine, and treatment was rapidly discontinued. Patient 2 was treated with clotiapine for 9 months; doses were titrated from 20 mg/day to 70 mg/day, with an improvement in the reported delusions, which also enhanced compliance with psychological and nutritional interventions. EKG, QTc, white blood count, and red blood count were not relevantly influenced by the introduction of clotiapine in either patient. No extrapyramidal effect was documented. These reports stress the need for further studies assessing the tolerability and potential effect of clotiapine in treating adolescents with AN and delusional symptomatology.

## 1. Introduction

Anorexia nervosa (AN) is an emerging psychiatric condition characterized by the refusal to maintain body weight at or above the minimal normal weight for age and height, intense fear of gaining weight, and disturbance in the experience of body shape [1]. AN usually begins during adolescence and occurs most commonly in females [2]. A restriction type (AN-R), marked by food restriction and commonly overexercising, has been distinguished from a binge-eating/purging type (AN-BP), where affected individuals eat large amounts of food in a relatively short time (binge eating), yet remain underweight or engage in behaviors to counteract weight gains, such as self-induced vomiting or use of laxatives or diuretics (“purging”) [3]. Beyond these core symptoms, the clinical course of AN is frequently influenced by numerous mental health and medical comorbidities, such as mood and anxiety disorders, personality disorders, autism spectrum disorder, and cardiovascular diseases [4,5,6,7].

International guidelines for the management of AN recommend treatments based on a multidisciplinary approach, mainly nutritional and psychotherapeutic interventions associated with the treatment of medical and socio-familial complications. Medications are frequently used to treat AN symptoms, including eating disorder symptoms (eating attitudes, refusal of gaining weight, preoccupation with shape and weight, obsessions with food) and psychiatric symptoms (depression, anxiety, obsessions, and compulsions) [8]. However, no definitive medication intervention exists to date, and randomized controlled trials have been ambiguous at best [9]. It is also worth noting that, according to the National Institute for Clinical Excellence guidance for the recognition and treatment of eating disorders [10], medications should not be used as the sole treatment for AN. Recent guidelines suggest the use of some antipsychotic medications in the management of low-weight children and adolescents with AN, but data regarding the efficacy and tolerability of these molecules are still scarce [11].

Among antipsychotic medications, the first generation or so-called “typical” antipsychotics, which include chlorpromazine and haloperidol, have been diffusely used in the past, whereas at present, their use is mainly limited to the management of psychotic disorders and episodes of psychomotor agitation. These medications can have severe side effects, such as drug-induced parkinsonism or tardive dyskinesia. Compared with the first generation of antipsychotics, “atypical” neuroleptics have fewer extrapyramidal side effects. The atypical antipsychotic most frequently studied in AN is olanzapine. The use of quetiapine and risperidone has also been documented [6,9,10].

Clotiapine, a dibenzothiazepine, is an atypical antipsychotic currently used in acute psychotic disorders, especially when they are associated with agitated or violent behavior. Clotiapine has been used in acute psychiatric emergencies since the late 1960s. It has a rapid onset of action and a strong sedative effect. It is indicated for the management of acute psychosis or exacerbations of chronic schizophrenia, manic episodes, other forms of acute psychotic disorders, agitation due to endogenous or exogenous (drugs, alcohol) causes, panic, inner uneasiness, drug withdrawal symptoms, states of depersonalization, hyperactivity, and sleep disorders [12]. The action of clotiapine is mainly exerted via downregulation of cortical 5HT2 receptors and blockage of 5HT3 receptors; a high affinity for 5HT6 and 5HT7 receptors has also been reported in the literature [11]. Its ratio of D2 to 5HT2 blockade is similar to that of clozapine and in the rat retinal model, clotiapine seems to act as an antagonist of the D4 receptor [11]. An oral form (40 mg) and an injectable form (10 mg) of clotiapine for intramuscular or intravenous use are currently available. The dose for acute psychosis is usually between 120 mg and 200 mg/day but can be as high as 360 mg/day [11]. Possible adverse effects are drug-induced movement disorders, significant initial tiredness and constipation, skin rashes, insomnia, headache, and facial sweating. Seizures, palpitations, and pain at the site of injection can also occur [11].

To our knowledge, the current scientific literature lacks any evidence concerning the use of clotiapine in the management of AN. In this study, we report the use of adjunctive clotiapine and its tolerability in the treatment of two adolescents affected by AN.

## 2. Materials and Methods

This study is a retrospective chart review of patients who were assessed between 1 January 2016 and 31 December 2019 in the Regional Centre for Feeding and Eating Disorders in children and adolescents in Bologna, Italy. Inclusion criteria were (a) a diagnosis of anorexia nervosa according to the Diagnostic and Statistical Manual of Mental Disorders—fifth edition (DSM-5) criteria [2]; (b) treatment with an antipsychotic during hospitalization; (c) acquisition of informed consent. Patients without complete clinical documentation were excluded from the study. Demographic and clinical data were obtained for patients treated with clotiapine. Diagnoses of AN were performed by clinicians trained in the field of ED. Specific subtypes of AN (AN-BP, AN-R, or atypical AN) were also assessed according to DSM-5 criteria. The Eating Disorder Inventory-3 (EDI-3) was administered to all patients to support the diagnostic process [12]. The patients were assessed by child neuropsychiatrists and screened for possible psychiatric comorbidities. To do so, the patients received, at admission, a complete psychiatric evaluation and were administered the Self Administrated Psychiatric Scales for Children and Adolescents (SAFA), a validated psychometric instrument used to assess psychiatric comorbidities in children and adolescents with eating disorders [13]. Concerning the treatment with clotiapine, we recorded: the length of treatment, the dosage at introduction, the maximum dosage, the frequency of dosing (once daily (OD), twice daily (BDS), three times per day (TDS)). Reasons for the administration and the interruption of the treatment, if it became necessary, were documented as well. Clinical evidence of adverse effect reactions (ADR) was also recorded. Plasma levels of red blood cells and white blood cells were recorded before and after the start of clotiapine. The results of electrocardiograms (EKG) before and after treatment introduction were reported as well. These EKGs were examined by pediatric cardiologists. All the psychopharmacological treatments, which were administered concurrently with clotiapine, were noted. The body mass index (BMI) of the patients at hospital admission and discharge was recorded.

## 3. Results

### 3.1. Characteristics of the Included Patients

During the selected period, 139 patients with AN and a mean age of 14.8 (+/−2.0) years were treated with antipsychotics. Two of them were treated with clotiapine. A thorough description of their history, symptoms, and management is presented below. Table 1 reports the tolerability of clotiapine in these two patients in terms of clinical, laboratory, and cardiological modifications.

### 3.2. Case 1

Our patient developed AN symptoms at the age of seventeen after undergoing thyroid surgery and radionuclide therapy due to a diagnosis of thyroid carcinoma. Within 5 months she progressively began to decrease her food intake and to manifest physical overactivity to drop weight, losing about 20 kg (going from 60 to 41.4 kg and 14.3 kg/m^2^) with cessation of menstrual periods. Mental status examinations revealed an intense fear of gaining weight, associated with an altered perception of her body image. A diagnosis of AN-R was made. For her altered eating behavior and metabolic status, the patient underwent multiple hospitalizations and day-hospital interventions. Antidepressant treatment with sertraline was started, later switched to fluoxetine. An antipsychotic treatment with aripiprazole later switched to olanzapine, was also started.

At the age of seventeen, during an outpatient visit at our center, new delusions emerged at the mental status examination. The patient expressed fixed ideas such as “smells contain calories” and “I cannot touch animals that have recently eaten, since this would influence my body weight”. These delusions had been negatively influencing the eating behavior of the patient for more than one month. Her weight was 44.9 kg and her BMI was 15.4 kg/m^2^. Oral clotiapine was introduced in therapy at the initial dosage of 10 mg/day, to reduce the delusional symptomatology. Concurrently, the patient was taking olanzapine 7.5 mg/day and sertraline 75 mg/day. Blood tests and EKG performed before the introduction of clotiapine are shown in the table.

A few minutes following the assumption of clotiapine, our patient presented a presyncope, characterized by partial loss of consciousness. After this event, the patient was admitted to our ward. Medical examinations revealed a blood pressure of 75/40 mmHg. Her skin was pale and her hands and feet cold. Blood exams at admission revealed a WBC of 3.8 × 10^3^/mmc (normal range 3.6–10.5) and an RBC of 4.2 × 10^6^/mmc (normal range 3.9–5.2). An EKG was performed at admission, which excluded arrhythmias and showed a sinus rhythm (60 bpm), with a corrected QT interval (QTc) of 415 msec. No extrapyramidal side effect was documented. Due to the persistence of discomfort and fatigue, 3 days after its introduction, the treatment with clotiapine was suspended and replaced by risperidone at the dosage of 1 mg/day, with a resolution of asthenia.

### 3.3. Case 2

Patient 2 started manifesting eating disorder symptoms at the age of sixteen when she began to avoid highly caloric foods and to manifest social isolation. Four months after the onset of these first symptoms, she started refusing to eat almost anything except salads. Bizarre thoughts concerning food and calories influenced her eating behavior significantly more than her desire to reach a low body weight. Physical hyperactivity in the form of refusal to sit and compulsively persistent standing position was more evident than overexercising. Therefore, she was diagnosed with atypical AN and underwent multiple hospitalizations due to her impaired metabolic status. Multiple pharmacological treatments were attempted, with antidepressants (sertraline, then switched to fluvoxamine), antipsychotic (olanzapine, aripiprazole, pimozide, quetiapine, haloperidol), and mood stabilizers (valproate).

At the age of eighteen, the patient was admitted to our ward for severe malnutrition (weight: 39.0 kg; BMI: 12.3 kg/m^2^). She appeared distrustful with the hospital staff, did not demonstrate curiosity in knowing or socializing with other patients, and refused to participate in the activities of the ward (school, afternoon activities, and groups)—behaviors that stressed her difficulty in interpersonal relationships. She showed intense anguish for every little change, spending her days in places that she had chosen and organized, unable to enter hospital rooms or places reserved for patients or to perform routine actions. Her mental status examination revealed severe obsessions and bizarre delusions, such as “everything I eat can set me on fire and melt me”. The patient was resistant to hygiene measures (washing, clipping her nails, changing clothes), refused to touch objects, eat and drink, and showed visible anxiety when small changes in her routine were suggested.

Clinical exams such as blood sampling or EKG were experienced as unbearably intrusive. She progressively refused to sit down and spent hours standing still beside her bed, which resulted in severe lower limb oedemas. Blood tests and EKG performed before the introduction of clotiapine are shown in Table 1. Psychopharmacological treatments were started, with sertraline (100 mg/day) and aripiprazole. Due to lack of efficacy, aripiprazole was switched to oral clotiapine, starting from 20 mg/day, up to 70 mg/day. Clotiapine was then maintained at this dosage, concurrently with sertraline 100 mg/day. The blood tests and EKG performed after the introduction of clotiapine are reported in Table 1. An EKG revealed persistence of sinus bradycardia (46 bpm); these EKG findings were stable 5 months after the introduction of clotiapine, with evidence of sinus bradycardia (54 bpm) and a QTc of 371 msec. No sedative effect was documented at any treatment dosage. No extrapyramidal side effect was documented. In response to this change in medical treatment, the delusional symptomatology reported at admission progressively, mildly decreased, making psychological and nutritional interventions with nasogastric tube feeding (NGT) possible. However, any attempt to increase the infusion rate was dropped due to the patient’s distress. The patient partially restored proper hygiene habits, accepting to wash once per week, but only in the presence of her parents. Despite refusing to change her clothes, she permitted her parents to wash them periodically. She increased her communication with her parents, rejecting clinical decisions with which she did not agree. The compulsive standing reported at admission progressively decreased but was not fully resolved. From a dietary point of view, however, no relevant improvement was noticed: the girl persisted in food refusal with the continued need of enteral nutrition through NGT. Treatment with clotiapine was maintained for 9 months with no evidence of occurring side effects. After 11 months of hospitalization, given the length of the hospital stay, the numerous previous hospitalizations, and the relatively improved compliance, the decision to continue medical, nutritional, and psychological interventions at home was made. At discharge from the hospital, her weight was 40.8 kg, with a BMI of 12.9. The patient was then discharged and transferred to territorial services. Follow-ups at our center continued for nearly two months, with the patient maintaining the discharge conditions.

## 4. Discussion

While the use of Clotiapine in the management of acute psychotic disorders such as schizophrenia is well documented, no evidence exists in the literature of its use in the treatment of psychiatric symptoms linked to eating disorders. With this study, we share our clinical data concerning the treatment with this drug of two young individuals with AN.

In the first case described above, the decision to use clotiapine was made after the onset of bizarre delusions, such as the belief that smelling certain things and touching animals that had eaten shortly before would affect personal body weight. We could not evaluate the possible effects of clotiapine on the patient’s symptoms because of the occurrence of a presyncope after administration and the persistence of fatigue until the treatment was interrupted, three days later. No direct evidence of EKG change in a pro-arrhythmic direction was documented. In the literature, among the possible side effects of clotiapine, significant initial tiredness is described [11]. We did not find previous evidence in the scientific literature of presyncope, or syncopal events directly linked to treatments with clotiapine. Orthostatic hypotension, however, represents a common adverse effect of antipsychotics [14]. Moreover, orthostatic hypotension and syncopal episodes represent a common feature of AN [15], especially in malnourished and dehydrated individuals. It is not clear whether the presyncope episode in our patient is or is not directly linked to the administration of clotiapine. Therefore, specific studies assessing the tolerability profile of this drug are needed.

In the second case described above, the administration of clotiapine did not trigger any side effects. The choice to use this drug resulted from the worsening of the obsessive and fixed delirious traits that the patient manifested, for example, her refusal to sit down (causing severe lower limb edema), to touch objects, to drink and eat, to wash, or change clothes. In this second case, once the pharmacological treatment was started, our patient began to improve her hygienic measures and to sit down, which resulted in an overall improvement of her general health status. Unfortunately, there was no change in her unwillingness to eat, and feeding via nasal feeding tube continued.

The literature provides conflicting evidence on the treatment with antipsychotics in children and adolescents with AN [16]. The rationale for the use of these drugs is based on studies documenting the involvement of the serotonergic and dopaminergic systems in the pathophysiology of AN [17]. Since they influence these circuits, atypical antipsychotics could play a role in the treatment of AN [17]. Some authors have hypothesized that AAP may improve the management of individuals with AN, postulating that the irrational thoughts regarding one’s body image may be connected with the delusions manifesting in psychotic disorders [16]. AAP could also influence anxiety and depressive symptoms in AN, and some of the known side effects of AAP, such as weight gain, could be beneficial in specific ED [17,18]. Yes, systematic reviews and metanalyses in this field have shown a few promising results, with some studies reporting no significant efficacy in improving the specific AN psychopathology or increasing weight gain [18,19]. Recent clinical guidelines on interventions for children and adolescents with ED only offer weak recommendations on the use of olanzapine and aripiprazole and indicate a lack of evidence for several different psychopharmacological treatments [20]. To our knowledge, the role of clotiapine in these patients has never been previously documented.

A relevant, specific pharmacodynamic characteristic of clotiapine is represented by its high affinity for serotonin receptors 5HT6 and 5HT7 [11]. The involvement of these receptors in the pathogenesis and maintenance of AN is still debated. A very recent study of Hrnjadovic and colleagues [21] tested 5-HT7 receptor involvement in the attentional set-shifting task (ASST) in rodents. ASST allows for the identification of several types of cognitive rigidity associated with neuropsychiatric conditions, including AN, schizophrenia, and mood disorders. The authors found that NMDA receptor blockade impaired ASST performance in rodents, which was reversed by administering the 5-HT7 receptor antagonist SB-269970. According to the researchers, these results indicate that 5-HT7 receptor mechanisms may influence the complex cognitive deficits identified in such psychiatric disorders as AN, schizophrenia, and depression, which are characterized by cognitive inflexibility and by increasing perseverative responses. Following these conclusions, we could postulate that the clinical improvement of acute psychiatric symptoms documented in the second of our cases could be partially attributed to the 5-HT7 blockade property of clotiapine. However, conflicting evidence exists on this subject. Previous research by Hinney and colleagues assessed the known Pro-279-Leu polymorphism in the 5-HT7 receptor gene by performing association allele frequencies tests and comparing 393 extremely obese children and adolescents, 142 underweight students, and 84 patients with AN. None of the association tests revealed nominal P-values below 0.3. The authors concluded that it is unlikely that the investigated polymorphism plays a major role in body weight regulation or AN [22].

Given the contradictory evidence for the involvement of the serotonergic 5HT7 receptors in the pathogenesis of core features of AN, we may not directly hypothesize a specific pharmacodynamic effect that would differentiate the effect of clotiapine from other atypical antipsychotics in treating this condition. Moreover, the emergence of a presyncope in one of our cases, requiring a precocious interruption of these pharmacological treatments, restricts the possibility of any clear-cut hypothesis. Moreover, the short follow-up period limits the chance to detect possible extrapyramidal side effects. These considerations and the specific nature of this study (a case report of two patients) should be acknowledged as limitations. Case reports represent a non-systematic description of the use of therapeutic interventions in real life. Nonetheless, their publication represents a widely used approach to reporting significant details of the use of specific drugs in uncommon populations or clinical settings, especially concerning the emergence of adverse drug reactions [23]. We believe that this report may represent a useful extension to the present literature on the use of antipsychotics in young individuals with AN, given the scarce literature on this population and the use of clotiapine in general.

## 5. Conclusions

Although it is not possible to draw reliable conclusions concerning the usefulness of clotiapine in the treatment of AN by describing its use in only two patients, with the present paper we intend to report first, the occurrence in patient 1 of a presyncope, which could be a side effect of the drug; second, the possible influence of clotiapine in the management of bizarre delusions inherent to ED, as in the case of patient 2. Given the overall improvement obtained in patient 2′s health conditions, with particular reference to her fixed obsessive and delusional attitudes, we postulate that the administration of clotiapine in adolescents with AN suffering from psychiatric complications could be an effective tool in their complex healing process. Further validation of this postulate is needed.

## Figures and Tables

**Table 1:** Laboratory and cardiological parameters before and after the introduction of clotiapine for the two patients.

	Patient 1		Patient 2	
	Before Clotiapine	After Clotiapine	Before Clotiapine	After Clotiapine
WBC	5.2 × 10^3^/mmc	3.8 × 10^3^/mmc	2.3 × 10^3^/mmc	3.82 × 10^3^/mmc
RBC	4.3 × 10^6^/mmc	4.2 × 10^6^/mmc	2.99 × 10^6^/mmc	3.79 × 10^6^/mmc
EKG	sinus bradycardia (55 bpm)	sinus rhythm (60 bpm)	sinus bradycardia (43 bpm)	sinus bradycardia (54 bpm)
QTc	430 msec	415 msec	381 msec	371 msec
EPS	None	None	None	None
Other ADR	/	Presyncopal episode	/	None

Abbreviations: ADR: adverse drug reactions; bpm: beats per minute; EKG: electrocardiogram; EPS: extrapyramidal symptoms; QTc: Corrected QT Interval; RBC: red blood cell count; WBC: white blood cell count.

## Data Availability

The datasets used and analyzed are available from the corresponding author on reasonable request.
